# Supplementary material for: COZOID: contact zone identifier for visual analysis of protein-protein interactions
Source: BMC Bioinformatics. 2018 Apr 6;19:125. doi: 10.1186/s12859-018-2113-6 (PMC5889581; doi:10.1186/s12859-018-2113-6)
Supplement: Supplementary file 2 — Software build. Executable binary file of the software tool. (ZIP 118,784 kb) [file 12859_2018_2113_MOESM2_ESM.zip › cozoid/java/docs/beansbinding-1.2.1-doc.zip!/org/jdesktop/el/ValueExpression.html]

ValueExpression (Beans Binding)


---


|  |  |  |
| --- | --- | --- |
| **Overview** \| **Package** \| **Class** \| **Use** \| **Tree** \| **Deprecated** \| **Index** \| **Help** | |  |
| **PREV CLASS**   **NEXT CLASS** | **FRAMES**    **NO FRAMES**     **All Classes** |  |
| SUMMARY: NESTED \| FIELD \| CONSTR \| METHOD | DETAIL: FIELD \| CONSTR \| METHOD |  |


---


## org.jdesktop.el Class ValueExpression

```
java.lang.Object
  org.jdesktop.el.Expression
      org.jdesktop.el.ValueExpression
```

**All Implemented Interfaces:**: java.io.Serializable

**Direct Known Subclasses:**: ValueExpressionImpl, ValueExpressionLiteral

---

``` public abstract class ValueExpression extends Expression ```

An `Expression` that can get or set a value.

In previous incarnations of this API, expressions could only be
read. `ValueExpression` objects can now be used both to
retrieve a value and to set a value. Expressions that can have a value
set on them are referred to as l-value expressions. Those that
cannot are referred to as r-value expressions. Not all r-value expressions
can be used as l-value expressions (e.g. `"${1+1}"` or
`"${firstName} ${lastName}"`). See the EL Specification for
details. Expressions that cannot be used as l-values must always
return `true` from `isReadOnly()`.

The `ExpressionFactory.createValueExpression(org.jdesktop.el.ELContext, java.lang.String, java.lang.Class )` method
can be used to parse an expression string and return a concrete instance
of `ValueExpression` that encapsulates the parsed expression.
The `FunctionMapper` is used at parse time, not evaluation time,
so one is not needed to evaluate an expression using this class.
However, the `ELContext` is needed at evaluation time.

The `getValue(org.jdesktop.el.ELContext)`, `setValue(org.jdesktop.el.ELContext, java.lang.Object)`, `isReadOnly(org.jdesktop.el.ELContext)` and
`getType(org.jdesktop.el.ELContext)` methods will evaluate the expression each time they are
called. The `ELResolver` in the `ELContext` is used to
resolve the top-level variables and to determine the behavior of the
`.` and `[]` operators. For any of the four methods,
the `ELResolver.getValue(org.jdesktop.el.ELContext, java.lang.Object, java.lang.Object)` method is used to resolve all properties
up to but excluding the last one. This provides the `base`
object. At the last resolution, the `ValueExpression` will
call the corresponding `ELResolver.getValue(org.jdesktop.el.ELContext, java.lang.Object, java.lang.Object)`,
`ELResolver.setValue(org.jdesktop.el.ELContext, java.lang.Object, java.lang.Object, java.lang.Object)`, `ELResolver.isReadOnly(org.jdesktop.el.ELContext, java.lang.Object, java.lang.Object)` or
`ELResolver.getType(org.jdesktop.el.ELContext, java.lang.Object, java.lang.Object)` method, depending on which was called on
the `ValueExpression`.

See the notes about comparison, serialization and immutability in
the `Expression` javadocs.

**Since:**
:   JSP 2.1

**See Also:**: `ELResolver`, `Expression`, `ExpressionFactory`, Serialized Form

---

**Nested Class Summary**

**Nested classes/interfaces inherited from class org.jdesktop.el.Expression**

`Expression.ResolvedProperty, Expression.Result`


**Constructor Summary**

`ValueExpression()`


| **Method Summary** | |
| --- | --- |
| `abstract  java.lang.Class<?>` | `getExpectedType()`             Returns the type the result of the expression will be coerced to after evaluation. |
| `Expression.Result` | `getResult(ELContext context, boolean trackResolvedObjects)` |
| `java.lang.Object` | `getSource()`             Returns the source of the expression. |
| `abstract  java.lang.Class<?>` | `getType(ELContext context)`             Evaluates the expression relative to the provided context, and returns the most general type that is acceptable for an object to be passed as the `value` parameter in a future call to the `setValue(org.jdesktop.el.ELContext, java.lang.Object)` method. |
| `abstract  java.lang.Object` | `getValue(ELContext context)`             Evaluates the expression relative to the provided context, and returns the resulting value. |
| `abstract  boolean` | `isReadOnly(ELContext context)`             Evaluates the expression relative to the provided context, and returns `true` if a call to `setValue(org.jdesktop.el.ELContext, java.lang.Object)` will always fail. |
| `void` | `setSource(java.lang.Object source)`             Sets the source of the expression. |
| `abstract  void` | `setValue(ELContext context, java.lang.Object value)`             Evaluates the expression relative to the provided context, and sets the result to the provided value. |

**Methods inherited from class org.jdesktop.el.Expression**

`equals, getExpressionString, hashCode, isLiteralText`

**Methods inherited from class java.lang.Object**

`clone, finalize, getClass, notify, notifyAll, toString, wait, wait, wait`

**Constructor Detail**

### ValueExpression

```
public ValueExpression()
```


**Method Detail**

### setSource

```
public void setSource(java.lang.Object source)
```

:   Sets the source of the expression. For ValueExpressions that have a
    source, any identifiers are evaluated relative to the source. For
    example, if the expression `"${first.name}"` has a source,
    then `"first"` is evaluated relative to the source.

    :   **Parameters:**: `source` - the initial source for identifiers; may be `null`

---


### getSource

```
public java.lang.Object getSource()
```

:   Returns the source of the expression.

    :   **Returns:**: the source of the expression

---


### getValue

```
public abstract java.lang.Object getValue(ELContext context)
```

:   Evaluates the expression relative to the provided context, and
    returns the resulting value.

    The resulting value is automatically coerced to the type
    returned by `getExpectedType()`, which was
    provided to the `ExpressionFactory` when this
    expression was created.

    :   **Parameters:**: `context` - The context of this evaluation. **Returns:**: The result of the expression evaluation. **Throws:**: `java.lang.NullPointerException` - if context is `null`.: `PropertyNotFoundException` - if one of the property resolutions failed because a specified variable or property does not exist or is not readable.: `ELException` - if an exception was thrown while performing property or variable resolution. The thrown exception must be included as the cause property of this exception, if available.

---


### getResult

```
public Expression.Result getResult(ELContext context,
                                   boolean trackResolvedObjects)
                            throws PropertyNotFoundException,
                                   ELException
```

:   **Throws:**: `PropertyNotFoundException`: `ELException`

---


### setValue

```
public abstract void setValue(ELContext context,
                              java.lang.Object value)
```

:   Evaluates the expression relative to the provided context, and
    sets the result to the provided value.

    :   **Parameters:**: `context` - The context of this evaluation.: `value` - The new value to be set. **Throws:**: `java.lang.NullPointerException` - if context is `null`.: `PropertyNotFoundException` - if one of the property resolutions failed because a specified variable or property does not exist or is not readable.: `PropertyNotWritableException` - if the final variable or property resolution failed because the specified variable or property is not writable.: `ELException` - if an exception was thrown while attempting to set the property or variable. The thrown exception must be included as the cause property of this exception, if available.

---


### isReadOnly

```
public abstract boolean isReadOnly(ELContext context)
```

:   Evaluates the expression relative to the provided context, and
    returns `true` if a call to `setValue(org.jdesktop.el.ELContext, java.lang.Object)` will
    always fail.

    :   **Parameters:**: `context` - The context of this evaluation. **Returns:**: `true` if the expression is read-only or `false` if not. **Throws:**: `java.lang.NullPointerException` - if context is `null`.: `PropertyNotFoundException` - if one of the property resolutions failed because a specified variable or property does not exist or is not readable.: `ELException` - if an exception was thrown while performing property or variable resolution. The thrown exception must be included as the cause property of this exception, if available. \* @throws NullPointerException if context is `null`

---


### getType

```
public abstract java.lang.Class<?> getType(ELContext context)
```

:   Evaluates the expression relative to the provided context, and
    returns the most general type that is acceptable for an object to be
    passed as the `value` parameter in a future call
    to the `setValue(org.jdesktop.el.ELContext, java.lang.Object)` method.

    This is not always the same as `getValue().getClass()`.
    For example, in the case of an expression that references an
    array element, the `getType` method will return the
    element type of the array, which might be a superclass of the type
    of the actual element that is currently in the specified
    array element.

    :   **Parameters:**: `context` - The context of this evaluation. **Returns:**: the most general acceptable type; otherwise undefined. **Throws:**: `java.lang.NullPointerException` - if context is `null`.: `PropertyNotFoundException` - if one of the property resolutions failed because a specified variable or property does not exist or is not readable.: `ELException` - if an exception was thrown while performing property or variable resolution. The thrown exception must be included as the cause property of this exception, if available.

---


### getExpectedType

```
public abstract java.lang.Class<?> getExpectedType()
```

:   Returns the type the result of the expression will be coerced to
    after evaluation.

    :   **Returns:**: the `expectedType` passed to the `ExpressionFactory.createValueExpression` method that created this `ValueExpression`.


---


|  |  |  |
| --- | --- | --- |
| **Overview** \| **Package** \| **Class** \| **Use** \| **Tree** \| **Deprecated** \| **Index** \| **Help** | |  |
| **PREV CLASS**   **NEXT CLASS** | **FRAMES**    **NO FRAMES**     **All Classes** |  |
| SUMMARY: NESTED \| FIELD \| CONSTR \| METHOD | DETAIL: FIELD \| CONSTR \| METHOD |  |


---
